# Cognitive Dysfunction After Analgesia and Sedation: Out of the Operating Room and Into the Pediatric Intensive Care Unit

**DOI:** 10.3389/fnbeh.2021.713668

**Published:** 2021-08-16

**Authors:** Ashley D. Turner, Travis Sullivan, Kurt Drury, Trevor A. Hall, Cydni N. Williams, Kristin P. Guilliams, Sarah Murphy, A. M. Iqbal O’Meara

**Affiliations:** ^1^Division of Pediatric Critical Care, Department of Pediatrics, Washington University in St. Louis, St. Louis, MO, United States; ^2^Department of Surgery, Virginia Commonwealth University School of Medicine, Richmond, VA, United States; ^3^Department of Pediatrics, Division of Pediatric Critical Care, Doernbecher Children’s Hospital, Oregon Health & Science University, Portland, OR, United States; ^4^Department of Pediatrics, Division of Pediatric Psychology, Pediatric Critical Care and Neurotrauma Recovery Program, Doernbecher Children’s Hospital, Oregon Health & Science University, Portland, OR, United States; ^5^Division of Pediatric Neurology, Department of Neurology, Washington University in St. Louis, St. Louis, MO, United States; ^6^Division of Neuroradiology, Mallinckrodt Institute of Radiology, Washington University in St. Louis, St. Louis, MO, United States; ^7^Department of Pediatrics, Massachusetts General Hospital, Harvard Medical School, Boston, MA, United States; ^8^Department of Pediatrics, Child Health Research Institute, Children’s Hospital of Richmond at Virginia Commonwealth University School of Medicine, Richmond, VA, United States; ^9^Department of Pediatrics, Uniformed Services University of the Health Sciences, Bethesda, MD, United States

**Keywords:** sedative neurotoxicity, pediatric intensive care outcomes, pediatric post-intensive care syndrome, cognitive dysfunction, delirium, opioid, benzodiazepine, neurodevelopment

## Abstract

In the midst of concerns for potential neurodevelopmental effects after surgical anesthesia, there is a growing awareness that children who require sedation during critical illness are susceptible to neurologic dysfunctions collectively termed pediatric post-intensive care syndrome, or PICS-p. In contrast to healthy children undergoing elective surgery, critically ill children are subject to inordinate neurologic stress or injury and need to be considered separately. Despite recognition of PICS-p, inconsistency in techniques and timing of post-discharge assessments continues to be a significant barrier to understanding the specific role of sedation in later cognitive dysfunction. Nonetheless, available pediatric studies that account for analgesia and sedation consistently identify sedative and opioid analgesic exposures as risk factors for both in-hospital delirium and post-discharge neurologic sequelae. Clinical observations are supported by animal models showing neuroinflammation, increased neuronal death, dysmyelination, and altered synaptic plasticity and neurotransmission. Additionally, intensive care sedation also contributes to sleep disruption, an important and overlooked variable during acute illness and post-discharge recovery. Because analgesia and sedation are potentially modifiable, understanding the underlying mechanisms could transform sedation strategies to improve outcomes. To move the needle on this, prospective clinical studies would benefit from cohesion with regard to datasets and core outcome assessments, including sleep quality. Analyses should also account for the wide range of diagnoses, heterogeneity of this population, and the dynamic nature of neurodevelopment in age cohorts. Much of the related preclinical evidence has been studied in comparatively brief anesthetic exposures in healthy animals during infancy and is not generalizable to critically ill children. Thus, complementary animal models that more accurately “reverse translate” critical illness paradigms and the effect of analgesia and sedation on neuropathology and functional outcomes are needed. This review explores the interactive role of sedatives and the neurologic vulnerability of critically ill children as it pertains to survivorship and functional outcomes, which is the next frontier in pediatric intensive care.

## Introduction

Concerns for adverse neurologic effects after surgical anesthesia in young children prompted a Safety Communication from the United States Food and Drug Administration in 2016 warning against the prolonged or repeated use of general anesthesia in children under the age of 3 years and pregnant women (United States Food and Drug Administration Safety Communication (FDA), 2019). This warning came after several retrospective studies reported learning disabilities and lower standardized test scores in children who underwent elective surgery as infants or toddlers (DiMaggio et al., 2011, 2012; Flick et al., 2011; Block et al., 2012), and experiments of anesthesia in young animals found increased neuronal death and abnormal synaptic plasticity to explain this cognitive dysfunction (Sanders et al., 2013; Jevtovic-Todorovic, 2018; Maloney et al., 2018). More recently, however, larger prospective studies of isolated anesthesia during infancy have not found a clear effect on cognition, though assessments may be limited by age-appropriate metrics in very young children (Sun et al., 2016; Warner et al., 2018; McCann et al., 2019). Interestingly, scrutiny of those earlier studies finds that cognitive effects were more significantly associated with prolonged or repeated anesthesias, or in infants with neurologic risk factors. That being the case, while children undergoing elective surgery are generally well, critically ill children are not healthy, and are subject to a number of metabolic and physiologic disturbances that render them uniquely neurologically vulnerable.

In pediatric intensive care units (PICUs) across the world, hundreds of thousands of children are necessarily treated with analgesics and sedatives pharmacologically similar to surgical anesthesia for prolonged periods during the neurologic stress of critical illness. Unlike anesthesia, which briefly overwhelms neurotransmission to facilitate invasive surgery, intensive care sedation alters similar neurotransmitter pathways but is titrated over time and allows for receptor adaptation. This may be of much greater consequence, particularly if this receptor adaptation occurs during sensitive periods of neurodevelopment. Pharmacologic opioids can interfere with the activity of endogenous opioid receptors, and a number of sedatives augment the activity of the gamma aminobutyric acid (GABA) receptor or inhibit signaling at the N-methyl-D-aspartate receptor (NMDAR). These receptors have important roles in normal neurodevelopment, and there is robust evidence from mammalian models that exogenous opioids, benzodiazepines, propofol, and ketamine affect neurogenesis, synaptogenesis, and myelination. Furthermore, prolonged multidrug analgesia and sedation may induce or exacerbate cerebral effects of critical illness such as neuroinflammation. In contrast to the seemingly divergent laboratory and clinical evidence for anesthetic neurotoxicity in healthy infants and toddlers, many young children who survive critical illness are recognized to have developmental delays and cognitive and psychological sequelae which are collectively termed pediatric post-intensive care syndrome (PICS-p) (Pinto et al., 2017; Manning et al., 2018; Watson et al., 2018; Choong, 2019). While critical illness is a complex paradigm when it comes to directly linking causative factors to post-discharge cognitive outcomes, multivariate analyses from adults and children consistently identify intensive care sedation and opioid analgesia as important and independent contributors (van Zellem et al., 2014; Watson et al., 2019; Dervan et al., 2020a; Ely, 2020).

## Cognitive Dysfunction

### Survivors of Pediatric Critical Illness or Injury Do Not Resemble the Typical Anesthesia Population

There is an established research literature focused on cognitive dysfunction after surgical anesthesia; results from this body of work should be cautiously applied to the pediatric intensive care population if at all. While there is disparate evidence of cognitive dysfunction after early childhood surgical anesthesia, there is a growing body of evidence that survivors of pediatric critical illness experience issues with cognition, particularly when a wide range of cognitive domains are considered. Unfortunately, many PICU outcomes studies have not prospectively collected sedation data or performed sub-analyses of sedative and analgesic contributions, and much is extrapolated from adult research. Anesthetic neurotoxicity necessarily regards sedative effects on healthy children with presumed normality in their developing brains. Children undergoing elective anesthesia are pre-screened for health and therefore differ considerably from critically ill children who suffer a range of unplanned homeostatic disruptions such as hypoperfusion, hypoxemia, acidosis, catabolic metabolism, and inflammation. Therefore, intensive care sedative neurotoxicity must also consider the synergistic impact of pharmacologically similar drugs on a wide array of neurologic stressors and injury in the developing brains of young children.

As understanding of PICS-p and interest in characterizing the long-term morbidities of PICU survivors has grown, so has the need to advance the field of outcomes measurement research in children (Manning et al., 2018). Although most cognitive outcome studies to date have focused on traumatic brain injury (TBI), congenital heart disease, and extracorporeal membrane oxygenation (ECMO), emerging literature shows that PICU patients who survive a wide range of diseases not typically considered to be primarily neurologic, including sepsis and respiratory failure, have cognitive dysfunction after hospital discharge (Bone et al., 2014; Kachmar et al., 2018; Williams et al., 2019). The PICS-p framework recognizes the fluid interdependence of a child’s cognitive outcomes on their physical, emotional, and social outcomes, and vice versa (Manning et al., 2018). As such, concerns have been raised about the use of specific drugs in the PICU, such as benzodiazepines and opioids, and the dose-dependence of effects on outcomes (Dervan et al., 2020a). van Zellem et al. (2014) reported lower overall intelligence quotient (IQ) scores in meningococcemia survivors that received any opioid compared to children that received sedation without opioid. Kachmar et al.’s (2018) systematic review found that when compared to healthy controls or normative population data, PICU patients treated with opioids or sedatives scored worse after discharge across the cognitive domains of attention, processing speed, executive functioning, memory, visual motor integration, and motor development. While a link between sedation and cognitive outcomes was not explored in their small case series, Elison et al. (2008) reported impairments in spatial and verbal memory, visual processing, pattern recognition, and processing speed in children admitted to intensive care for sepsis, sickle cell anemia crisis, or respiratory failure. Dysfunction in any of the aforementioned domains has the potential to yield a negative impact on children at home, in the community, and within learning environments.

Having a multidimensional perspective on what constitutes cognitive outcomes is important and relevant in critical care, as not all domains may be affected to the same degree nor recover at the same rate. Not only is PICS-p multifaceted, but the cognitive domain within the framework is also incredibly complex as well. If cognition is the “process of acquiring, understanding, and using information”, then academic performance may be a sensitive, if not always specific, metric for cognitive dysfunction in school aged children and teens. In a prospective multicenter trial, Watson and Curley followed children after PICU discharge, and found that while 6 month Pediatric Cerebral Performance Category (PCPC) assessment of cognitive function was decreased in only 9–15% of patients 6 months after discharge, nearly 40% of survivors self-reported impaired school functioning on the Pediatric Quality of Life Inventory, highlighting the importance of cognition in action. Indeed, school performance is impacted by specific cognitive processes like those described above (e.g., processing speed, working memory, general memory, visual motor integration, etc.), but it is also subject to physical issues like poorly controlled pain and sleep deprivation, as well as issues related to mood disturbance, motivation, and ability to pay attention. When Madderom et al. (2016) and Schiller et al. (2016) followed cognitive outcomes in ECMO survivors over 8 years they found that while gross measures of intelligence were within normal ranges, these patients required extra help in school at a higher proportion than the general population, and specifically struggled with things such as selective attention. PICS-p patients also commonly report feeling as though they cannot keep up with their peers, have difficulty focusing and paying attention, or describe their thinking as slow or clouded. Therefore, it is important to separate testable cognition (e.g., neuropsychological direct assessment) from functional or social cognition (e.g., parent or teacher proxy, academic performance) as the field moves forward in unifying the structure and approach for assessing cognitive outcomes within the PICS-p framework. With this distinction in mind, clinicians and researchers should be mindful of what available outcomes tools actually measure in the realm of pediatric cognition, functional or adaptive skills, and development.

### Limitations With Current Approach on Measuring Cognitive Outcomes in PICU Survivors

**FIGURE 1:**
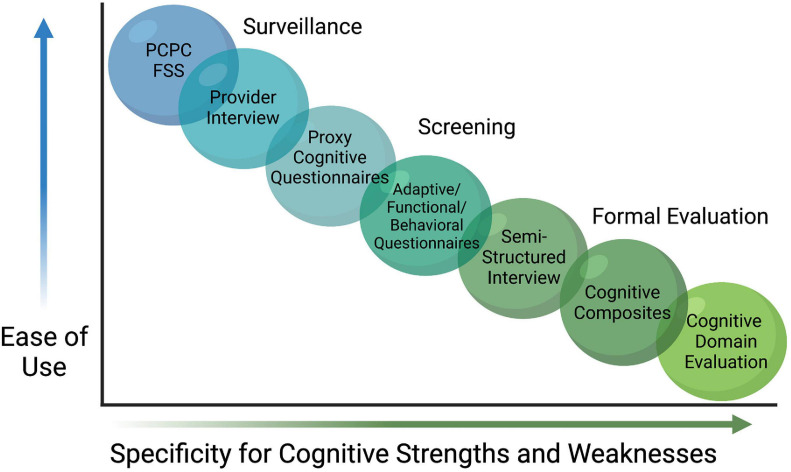
Continuum of Cognitive Assessments. A variety of assessment tools and approaches exist for evaluating cognitive dysfunction after discharge from pediatric intensive care. While some are useful for surveillance or screening, they are less specific for many aspects of cognitive function, particularly cognitive domains. Within the limitations of each strategy, investigators can select an approach that best addresses their aims. Pediatric Cerebral Performance Category (PCPC), Functional Status Scale (FSS).

With the inherent complexity of cognition comes variability on how cognitive outcomes are quantified in the research literature. Many studies have used gross outcome evaluation tools that broadly measure cognitive functioning while other studies indirectly assess cognitive function via variables such as need for academic help in school, Individualized Education Plans (IEPs), or a learning disability diagnosis. A recent scoping review identified 183 cognitive outcome studies using 115 different assessment tools (Maddux et al., 2020). In this review, various versions/revisions of the preschool and school-age versions of the Wechsler Intelligence Scales were most commonly used (*n* = 82 studies, 45%), followed by the PCPC (*n* = 36, 20%) and a wide array of additional measures to account for the remaining 35%. The variation in the assessment tools utilized to quantify cognitive outcomes likely explains some of the differences reported across studies, as it is difficult to compare direct measurements of cognition or intelligence with observational reports of performance. More uniform assessments across professional disciplines will be a crucial step forward in drawing conclusion from this research. Fortunately, work is underway that will leverage the findings from the scoping review mentioned above into a recommended core outcomes measure set (Fink et al., 2020). Even with progress in the field at large, any single set of measures is unlikely to meet the full need in pediatrics. As shown in Figure 1, many instruments are limited by age group and developmental stage (i.e., many tools are not appropriate for very young children who have not yet developed the cognitive flexibility of executive functioning), capacity, professional qualifications needed to administer, and a need for validation in a PICU population.

Gross measures and/or screeners of cognitive function are easier to administer, and often have “short form” versions that are less time-intensive and with fewer user restrictions than traditional neuropsychological measures. The PCPC, for example, can be retrospectively approximated as a baseline measurement and then compared before and after illness in order to identify changes in cognitive status on a 6-point scale that scores normal as a 1, moderate disability as a 3, and vegetative state as a 5 (Fiser, 1992). While the use of a measure like the PCPC is common (Schiller et al., 2016), and may be appropriate for many studies seeking to capture a general cognitive outcome variable, it does not yield specific outcomes germane to a PICU population when cognition is a primary outcome of interest. Similarly, seminal trials designed to detect adverse effects of anesthesia exposure in young children (PANDA (Sun et al., 2016), MASK (Warner et al., 2018), and GAS (McCann et al., 2019) trials) found no difference on overall IQ after single or multiple anesthetic exposures, though such broad assessments with cognitive composites like IQ may also lack the sensitivity to measure smaller but clinically meaningful changes within specific cognitive domains (Hrabok et al., 2014). As noted above, domains of attention, processing speed, executive functioning, memory, visual motor integration, motor development, verbal memory, visual processing, and pattern recognition are previously identified areas of vulnerability in PICU populations, with correlates to neuropathology in mammalian models, but cognitive domains require specific assessments often with trained professionals which may limit availability for large research studies.

Similarly, survey data obtained from parents or teachers are also used to approximate cognitive function, though this methodology introduces a layer of subjectivity that may limit interpretation. Observational reports do not directly or objectively measure cognition, but measure observable adaptive skills or behavior, and, as such, studies show proxy reports often differ substantially from formal neuropsychological evaluation of cognitive function, with outside factors confounding results (Kurowski et al., 2013; Toplak et al., 2013). Highlighting this point, a meta-analysis by Ing et al. (2021) included over 800 children who were prospectively enrolled in trials that collected neurodevelopmental data after a singular anesthetic exposure showed a 68% increased risk of reported “significant impairment” on the Behavior Rated Inventory of Executive Function by parent or caregiver (BRIEF, n.d.; Toplak et al., 2013) and a 47% increased risk of significant emotional/behavioral disturbances on the Child Behavior Checklist (CBCL; Achenbach, 2011; Achenbach and Rescorla, 2013). However, in this same meta-analysis there was no effect of anesthesia on formal evaluation of the Full-scale Intelligence Quotient (FSIQ).

The collective understanding of PICS-p and interest in characterizing the long-term cognitive morbidities of PICU survivors is relatively new. As such, methods related to outcomes measurement research in children, especially those focused on cognition in PICU survivors, remain vague and in need of refinement (Fink et al., 2020). Perhaps utilizing gross outcome evaluation tools and/or screeners that broadly measure cognitive functioning will yield data needed to ask the next phase questions. However, an understanding of different cognitive domains and how they are tested in outcomes research should be paired with knowledge of cellular level pathophysiology of the exposures of interest (e.g., sedatives and anesthetics) in order to generate and test hypotheses seeking to identify modifiable factors for improving cognitive outcomes. If we are to move the needle in regard to modifiable factors related to sedation-related post-PICU outcomes, it is imperative that a more domain specific/targeted approach with the right tools at the right time is used.

### Developmental Considerations Related to Measuring Cognitive Outcomes in PICU Survivors

While all of the limitations on measuring cognitive outcomes in PICU survivors discussed above are important, issues related to age and developmental stage are especially salient. As noted above, children in the PICU are treated with analgesics and sedatives for prolonged periods during the neurologic stress of critical illness during sensitive periods of neurodevelopment. Mammalian models show notable impact on neurogenesis, synaptogenesis, and myelination suggesting that younger children may be more vulnerable than older children to the effects of analgesics and sedatives based on how brains are built over time via the developmental process.

Timing of assessment and age are key considerations in evaluating pediatric patients for cognitive sequelae, as a study by Dervan et al. (2020a) of over 2,000 PICU patients identified age less than 2 years as a risk factor for worse PCPC score at PICU discharge. This is important given that injury to the brain or derailments in development during infancy and early childhood can have far reaching impacts due to this being a foundational period in brain development. Specifically, starting very early in development, cortical organization occurs through the rearranging of neurons in the supporting structure of the cortex and structural differentiation in the central nervous system occurs (i.e., neuronal differentiation, glial cell growth, and axonal and dendritic growth) (Volpe et al., 2017). These neurons engage in synapse formation by sending out axons to connect with both nearby and distant areas of the brain, setting the foundation (and growth trajectory) for circuitry connecting far reaching areas of the brain, which is essential for continued and normal development of long-term cognitive function (Volpe et al., 2017). Additionally, from birth to age 2 years, myelination and rapid synapse formation are the most active and important processes of cortical development (Kinney et al., 1988; Huttenlocher and Dabholkar, 1997). This all suggests that the timing of neurodevelopmental assessments may also be critical as survivors of childhood critical illness may “grow into” their cognitive deficits if foundational neurodevelopment is affected (Caprarola et al., 2017; Sobotka et al., 2018; Choong, 2019).

Adding to the complexity of timing, assessing cognitive outcomes in a younger population is challenging (based on the limitations of available instrumentation), which in-turn may contribute to an under-appreciation of the impact intensive care sedation has on children as they age. Infant developmental assessments are typically carried out up until 2 years of age; however, research focused on early life assessment in vulnerable populations suggests that this follow-up should continue at least through preschool age, and some argue through adolescence, to ensure that children with problems are being identified and are receiving appropriate intervention services (van de Weijer-Bergsma et al., 2008). The reasons for longitudinal follow-along are twofold: (1) the pediatric brain changes overtime and (2) the availability, reliability, and validity of tools for assessing a wide range of cognitive abilities dramatically increases for school aged (and beyond) populations. Pediatric survivors of critical illness may also be left with vulnerabilities that do not surface until significant stress is applied. Childhood and adolescence are full of increasingly difficult academic gradations and challenging life transitions that may unmask dysfunction at a later time, and ensuring resilience may also be part and parcel of improving post-intensive care outcomes. Stronger collaborations between cognitive experts (i.e., neuropsychologists) and PICU providers can impact early detection and support to engage in early intervention programs and cognitive rehabilitation services that will impact outcomes for children and their families following childhood critical illness throughout developmental stages and recovery.

### Delirium as Prodrome

**FIGURE 2:**
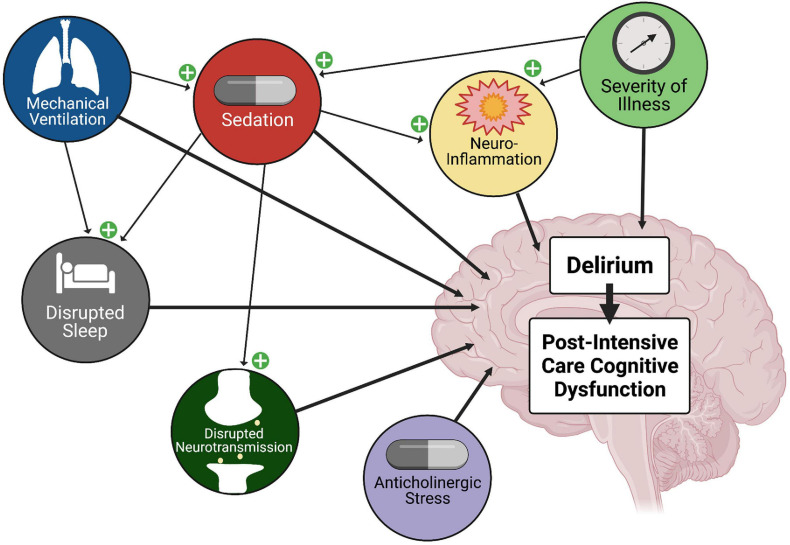
Factors Contributing to Delirium and Post-Intensive Care Cognitive Dysfunction. ICU delirium is multi-factorial in nature, and is associated with post-intensive care cognitive dysfunction. Mechanical ventilation, severity of illness, and anticholinergic burden are linked, and importantly, sedatives have been shown to disrupt sleep/wake cycles. In animal models, sedatives and opioid analgesics have been linked to neuroinflammation and altered connectivity and neurotransmission. Thus, treatment with sedatives and opioid analgesics has an integral role in the development of delirium and post-intensive care cognitive dysfunction.

Delirium is a neurobehavioral syndrome characterized by disrupted arousal, sleep-wake disturbances, altered sensorium, and cognitive dysfunction. Delirium is common in the PICU, and associated with prolonged hospitalization and increased mortality rates (Traube et al., 2017a,b). As shown in Figure 2, risk factors for delirium include length of stay, severity of illness, anticholinergic burden, sleep disturbance, ICU interventions such as mechanical ventilation, and aspects of sedation including medication choice and depth and duration of sedation (Turkel and Tavaré, 2003; Turkel et al., 2006). Animal models support many of these delirium variables including impaired cholinergic signaling, neuroinflammation, altered sleep/wake cycles, and disrupted neurotransmission (Flacker and Lipsitz, 1999a,b; Maldonado, 2018). Opioid analgesic and GABA and NMDA sedatives may contribute to delirium by altering neurotransmission, disrupting sleep architecture, potentiating neuroinflammation, inducing hyperalgesia, or inducing neuronal and glial cell dysfunction and/or death. Given this, it is not surprising that there are significant associations between intensive care sedation and opioid analgesia and delirium (Gaudreau et al., 2005).

Many risk factors for delirium are also risk factors for morbidity and mortality in the ICU, and making distinctions can be difficult (Wilson et al., 2020). Reality may well be a cyclic relationship: those with greater illness or injury severity are more susceptible to delirium given their underlying illness and increased exposure to known risk factors, and delirium in turn results in behavioral treatment with sedatives that contribute to brain injury and longer duration invasive mechanical ventilation. It is not always clear whether delirium is a treatable transitional phenomenon between critical illness brain stress and irreversible neurologic morbidity, but several studies support delirium as a harbinger of post-intensive care cognitive dysfunction (Mitchell et al., 2018). Girard et al. (2010) and Pandharipande et al. (2013) have reported associations between ICU delirium duration and cognitive dysfunction at 3 and 12 months post-discharge. Likewise, Morandi et al. (2012) not only affirmed this relationship between length of ICU delirium and cognitive dysfunction at 12 months post-discharge, but using magnetic resonance imaging found an underlying loss of white matter integrity in these patients. Interestingly, Hayhurst et al. (2020) looked at delirium subtypes and observed a greater likelihood of cognitive dysfunction in patients with hypoactive delirium than hyperactive delirium. Hyperactive delirium typically garners more attention at the bedside, as these patients can be challenging to manage behaviorally, and thus, hypoactive delirium may go undetected. This is of particular concern in the PICU because hypoactive delirium appears to be more closely linked with post-discharge cognitive dysfunction, and young children may have higher rates of hypoactive or mixed delirium (Traube et al., 2017a).

## Sleeping With One Eye Open

### Sleep Architecture Is Disrupted During Critical Illness

Intensive care sedation directly contributes to sleep disruption which is an additional factor leading to cognitive dysfunction following critical illness. Sleep is an essential biological function and, in children, it is critical for brain maturation and development. Early childhood sleep correlates with gray and white matter volumes particularly in the dorsolateral, prefrontal, and hippocampal cortex (Jan et al., 2010; Kocevska et al., 2017). Sleep disturbances during crucial periods of brain development may impact synaptic plasticity, neuronal migration, myelination, and memory consolidation leading to effects on cognition (Astill et al., 2012; Spruyt, 2019). In healthy children, shorter sleep is associated with worse cognitive function, specifically worse school performance and executive and multiple-domain cognitive functioning, and critically ill children are certainly prone to disrupted sleep (Astill et al., 2012; Spruyt, 2019). Polysomnography (PSG) in critically ill children demonstrates severe sleep fragmentation, alterations in sleep architecture, and circadian shifts with increased daytime sleep (Carno et al., 2004; Al-Samsam and Cullen, 2005; Armour et al., 2011; Kudchadkar et al., 2015). Multiple factors contribute to this sleep disruption, including non-modifiable factors, such as metabolic derangements of critical illness and necessary patient care interventions. However, modifiable factors are also an issue, including too little daytime light and orienting activity, excessive nighttime light and noise, patient care interventions that could safely be postponed, mode of mechanical ventilation, and the effect of medications including sedatives (Parthasarathy and Tobin, 2002; Al-Samsam and Cullen, 2005; Kudchadkar et al., 2014a,b; Greenfield et al., 2020).

**FIGURE 3:**
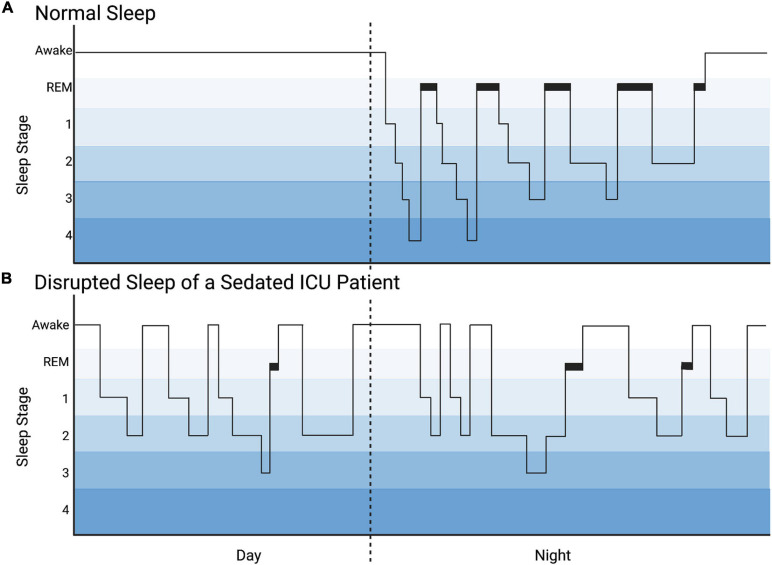
Hyponogram of Normal Sleep and Disrupted Sleep of a Sedated ICU Patient. **(A)** The hyponogram of normal sleep depicts the cyclic progression through sleep stages: non-rapid eye movement [divided into light sleep (stages 1, 2) and deep slow wave sleep (stages 3, 4)] and rapid eye movement (REM) sleep. **(B)** The hyponogram of a sedated intensive care unit (ICU) patient is noted to have sleep distributed during the day and night and sleep fragmentation depicted by frequent awakenings. This patient has a predominance of light sleep (stages 1, 2) and decreased deep slow wave sleep (stages 3, 4) and REM sleep.

While both sleep and sedation are characterized by decreased responsiveness to external stimuli, sleep and sedation are not equivalent. As shown in Figure 3, sleep is influenced by circadian rhythmicity and homeostatic control mechanisms and is characterized by cyclic progression of stages: non-rapid eye movement (NREM) [further divided into light sleep (Stages 1, 2) and deep slow wave sleep (Stages 3, 4)]; and rapid eye movement (REM). Achieving healthy sleep stage progression and adequate proportions of deep slow wave sleep and REM are essential to healthy brain development, maturation, and healing after injury. Certain associations with these vital sleep stages are particularly salient in patients with brain injury and/or critical illness brain stress. Slow wave sleep, measured by delta waves on electroencephalography (EEG), is essential to waste clearance through the glymphatic system including inflammatory cytokines, glutamine, lactate, and damage associated proteins (Xie et al., 2013). Slow wave sleep is also associated with reductions in brain temperature and metabolic demand for oxygen and glucose, as well as replenishment of ATP and glycogen stores (Aalling et al., 2018; Caporale et al., 2021). REM sleep deprivation decreases the threshold for cortical spreading depressions which are found frequently after acquired brain injuries and thought to contribute to secondary brain injury (De Vasconcelos et al., 2004; Levesque et al., 2021). Interestingly, a recent study of analgesia and sedation after a variety of brain injuries found that benzodiazepine treatment increased the incidence of cortical spreading depressions (Hertle et al., 2012).

### Intensive Care Analgesia and Sedation Alter Sleep Cycles

Although critically ill patients receiving sedatives and analgesics may appear asleep, PSG or EEG reveal significant fragmentation and alteration in sleep architecture which has been more heavily studied in adults than children (Figure 3; Armour et al., 2011; Weinhouse and Watson, 2011). Benzodiazepines decrease both restorative slow wave sleep and REM sleep while prolonging stage 2 NREM (Borbély et al., 1985; Achermann and Borbély, 1987; Pandharipande and Ely, 2006; Weinhouse and Watson, 2011). Similarly, propofol significantly decreases REM sleep (Kondili et al., 2012). Opioids also decrease slow wave and REM sleep, and increase stage 1 NREM (Cronin et al., 2001; Pandharipande and Ely, 2006). Dexmedetomidine, however, appears to more closely resemble natural sleep architecture on EEG in adults and has been shown to reduce sleep fragmentation, though there is an apparent increase in the proportion of stage 2 NREM sleep compared to deep slow wave and REM sleep (Huupponen et al., 2008; Alexopoulou et al., 2014; Akeju et al., 2016). Interestingly, a recent study by Dervan et al. (2020b) found that critically ill children receiving primarily combined opioid and dexmedetomidine sedation exhibited a loss of diurnal sleep variation and spent more time in stages 1 and 2 sleep than normal for age. Moreover, this study noted almost complete absence of REM sleep, and that sleep was so frequently interrupted as to be fragmented into periods averaging 25 min with an interquartile range of 14–36 min. Despite the potential for meaningful differences between adults and children, there is sparse information regarding the specific effects of sedative medications on sleep in the PICU, particularly in young children who are in the midst of developmentally regulated shifts in neurotransmitter and receptor concentrations. This must be further elucidated in order to adjust sedative regimens to minimize the deleterious effects on sleep. Strategies to increase the proportion and duration of deep slow wave sleep and REM sleep may be potential therapies to reduce secondary brain injury and morbidity. For example, daily interruptions of continuous sedation in adults have been shown to increase stages 3 4 NREM and REM sleep, and thus, may be considered to improve sleep of critically ill pediatric patients (Oto et al., 2011).

### Sleep Disturbances May Also Affect Post-discharge Recovery

Sleep disturbances persist for many children following resolution of critical illness. In a long-term follow up study of pediatric critical illness, survivors suffered significantly more sleep problems than healthy controls and 72% scored at high risk for sleep disorders (Als et al., 2015). Patients that survive neurocritical care with acquired brain injury are at particular risk of deleterious effects from sleep disorders, and over half report clinically significant sleep disturbances, many scoring more than two standard deviations worse than healthy peers, particularly in areas of initiation and maintenance of sleep (Williams et al., 2020). In studies of pediatric traumatic brain injury (TBI) survivors, sleep disturbances persist for years, and children with worse sleep had increased emotional and behavioral problems, including increased internalizing behaviors, externalizing behaviors, and executive function impairments (Beebe et al., 2007; Shay et al., 2014; Sandsmark et al., 2017; Luther et al., 2020). In contrast to the pediatric critical care and TBI literature, where data are incomplete for the link between sleep disturbances and neuropsychological assessments, there is robust data in healthy children showing a positive association between sleep duration and cognitive performance (Astill et al., 2012). Because critical illness by its very nature subjects the brain to intense metabolic and physiologic stress that may be injurious, the effect of sleep disturbances on cognitive functioning after pediatric critical illness must be further evaluated. Sedative use in the ICU contributes to sleep fragmentation and altered sleep architecture, which may further contribute to adverse cognitive outcomes; and while sedative exposure during acute illness is often the focus, many ICU patients require prolonged weaning from these drugs during convalescence and recovery. Additionally, the persistence of sleep disruption following pediatric ICU discharge should be considered a contributing and potentially modifiable factor in studies evaluating the link between sedatives and cognitive outcomes in these children.

### Sleep Tight, Don’t Let the Nighttime Bathtime Bite

Given the probable detriment of sleep disturbance on neurologic recovery after critical illness, sleep/wake patterns and sleep hygiene during critical illness and post-discharge convalescence are important considerations. Sleep promotion is not only likely to be therapeutic, but characterization of abnormal sleep architecture may add to diagnostic and/or prognostic paradigms. Unlike adult and neonatal ICUs, sleep promotion interventions are uncommon in many PICUs (Kudchadkar et al., 2014b). To promote restorative sleep, strategies should be implemented for increasing daytime natural light exposure while minimizing light at night, reducing nighttime noise, and avoiding noxious nocturnal patient care that could be safely postponed, such as baths, linen changes, and scheduled x-rays. Augmenting daytime activity, and early rehabilitation and mobility efforts with age-appropriate schedules for sleep, therapeutic activity, and napping can also promote circadian rhythm and sleep. Multi-disciplinary education on the potentially deleterious effects of analgesia and sedation on sleep is also warranted, along with accounting for these effects during daily care decisions. Advances are also needed in the measurement of sleep in critically ill children. Existing research focuses on the use of PSG and EEG to measure sleep stages and sleep disruption which are reliable but resource intensive, and more difficult to interpret in a critically ill population. Equipment is costly and not only does interpretation require qualified personnel, but delayed results limit actionable information. Moreover, because our understanding of sleep and pediatric critical illness is still in its infancy, specific interventions are lacking. To advance our understanding of ICU sleep, improved techniques for sleep monitoring are needed to promote accuracy and accessibility on a larger scale. This is of particular importance since routine sleep monitoring may be useful in neurologic prognostication following critical illness, with studies showing early return of sleep architecture to be associated with better outcomes (Arnaldi et al., 2016). To combat sleep disruption after PICU discharge, parental education and screening for sleep disruption should be incorporated into discharge teaching and follow up care for childhood critical illness survivors (Beebe et al., 2007; Shay et al., 2014; Als et al., 2015; Sandsmark et al., 2017; Luther et al., 2020; Williams et al., 2020). Longitudinal studies are also warranted to objectively study sleep cycles after critical illness to determine which sleep disorders are most prevalent and associated with poor outcomes, and which patients are most at risk. Finally, interventions to improve sleep during this critical period of recovery are needed, as no evidence based interventions currently exist in this vulnerable population. Optimizing sleep during critical illness and following discharge represents a potentially modifiable target to accelerate recovery and may be crucial to improving outcomes.

## Is a Better Mouse(Rat)Trap the Answer?

### Preclinical Models of Anesthetic Neurodevelopmental Toxicity Do Not Resemble the Typical PICU Population

Animal models have long been vital to developmental neuroscience research due to similarities in brain maturation that are preserved across species. Rodent models are particularly useful because the timeline for brain development in small mammals is compressed over the first weeks of their comparatively abbreviated life-span (Dobbing and Sands, 1979; Rice and Barone, 2000; Semple et al., 2013). Key benefits of preclinical models are that many variables can be controlled for in order to answer specific mechanistic questions, and brain tissue can be directly analyzed for pathology. Because critical illness brain stress and injury are complex, teasing apart the contribution of a particular factor such as sedation often requires a model species. Moreover, if a laboratory model “reverse translates” with enough fidelity so as to provide complementary cellular and molecular context to inform parallel clinical research, expedient, actionable data can be acquired to improve bedside practice.

To date, preclinical models of developmental sedative neurotoxicity have largely focused on the effects of single- and multi-drug anesthesia in healthy infant-approximate animals, as this neurodevelopmental period includes the brain growth spurt and the peak of synaptogenesis (Lu et al., 2006; Lunardi et al., 2010). Reduced synaptogenesis and increased neuronal death in animal models, in conjunction with clinical studies focused on infants and toddlers, may have inadvertently given the impression that only the infant and toddler brain is vulnerable, despite potential effects on later neural refinement. In fact, De Roo et al. (2009) and Briner et al. (2011) found an *opposite* age-dependent synaptogenic increase in rodents age-approximated to later childhood and adolescence after identical treatments, and too many inefficient synapses can be just as detrimental as too few. Nonetheless, the majority of this research has been performed in rodents and non-human primates with reproducible neuropathology and learning and memory deficits. Notably, many drugs that are used in the operating room are used in the PICU or are pharmacologically similar, yet there are few if any studies of the comparatively prolonged multi-drug exposures that occur during critical illness. In contrast to surgical anesthesia, intensive care sedation is administered in escalating doses for days to weeks, not including weaning during recovery. Moreover, many PICU patients also undergo invasive procedures under anesthesia. PICU-typical sedation is primarily opioid-based in order to ensure analgesia, and is commonly combined with GABA agonist benzodiazepines (Hartman et al., 2009; Curley et al., 2015). The alpha2 agonist sedative dexmedetomidine has seen increasing use in the last decade despite incomplete data regarding its effects on the developing brain (Thompson et al., 2019; Berkenbosch, 2020). Ketamine and barbiturates are used less frequently, and propofol is uncommon in pediatric intensive care compared to adult ICUs (Hartman et al., 2009; Curley et al., 2015).

### Common Analgesics and Sedatives Induce Neuropathologic Changes in the Mammalian Brain

Pre-clinical models demonstrate increased apoptotic and necrotic neuronal death in cell culture and infant-approximate animals after treatment with opioids, *N*-methyl-D-aspartate receptor (NMDAR) antagonists (ketamine, nitrous oxide, and inhalational -fluranes for example), and γ-aminobutyric acid (GABA) receptor agonists (benzodiazepines, barbiturates, propofol, etomidate, and inhalational -fluranes) (Hu et al., 2002; Young et al., 2005; Lu et al., 2006; Nikizad et al., 2007; Lunardi et al., 2010; Milanovic et al., 2017; Obradovic et al., 2018). Seminal work by Ikonomidou et al. (1989, 1999) showed that excitatory glutamate activity at the NMDAR plays a central role in neuronal survival, and NMDAR blockade may accelerate normal developmental neuronal pruning. Additionally, glutamate and GABA signaling regulate neuronal migration, axonal outgrowth, and synaptic plasticity (McDonald and Johnston, 1990; Komuro and Rakic, 1993; Luján et al., 2005). Opioid treatment of adult rats reduces excitatory glutamate synapses and increases GABA synapses in the hippocampus, as well as inhibiting glutamate release (Nugent et al., 2007; Cai et al., 2016). Taken together, inappropriate neuronal loss and stunted neural outgrowth and connectivity are plausible explanations for clinically observed cognitive dysfunction. Likewise, decreased synaptic density or signaling in infants and toddlers would impact cognition; and because synaptic plasticity is meant to be experiential-based, drug-increased synapses in older children may lack necessary integration and result in disorganized connectivity and neurotransmission. Age younger than 2 years is a risk factor for delirium and poor outcomes, and these age-dependent differences between too few neurons and synapses and too many synapses in laboratory studies may be reflected clinically in hypoactive versus hyperactive delirium, as hypoactive delirium appears to be more common in young children. Correlation of synaptic pathology with early delirium assessments in animal models would increase their translational value.

Because much of the aforementioned investigation has been performed in prenatal or infant-approximate animals during neurogenesis, gliogenesis, and synaptogenesis, there is little accounting for the peak of myelination or synaptic pruning and plasticity of later childhood. For example, Briner and DeRoo’s anesthetic work been replicated by Xu et al. (2019) in early childhood-approximate mice treated with midazolam for 5 days, affirming increased synaptic density at a time when experiential-based pruning should predominate. This effect may be purely neuronal, but synaptic plasticity is chaperoned by astrocytes and microglia, and drug effects in this regard have not been adequately characterized. There is experimental evidence for glial effects of sedatives, and additional targeted study of PICU-typical exposures is needed as glial cells (astrocytes, oligodendrocytes, and microglia) are not just profoundly important to neurodevelopment, they are vital for neurologic homeostasis and recovery, making them of particular import to intensive care delirium and neurologic outcomes. Inhalational anesthetics with NMDAR and GABA activity reduce the expression of astrocytic glial fibrillary acidic protein (GFAP) and disrupt astrocytic cytoarchitecture in culture, as well as limit the support of axonal outgrowth by astrocytes (Culley et al., 2013; Ryu et al., 2014). Similarly, Iqbal O’Meara et al. (2020) noted reduced GFAP after prolonged treatment of early-childhood approximate rats with benzodiazepine, opioid, or combined benzodiazepine and opioid. Pertinent to neurodevelopment and neurotransmission, GFAP anchors glutamate transporters necessary for balancing ambient glutamate levels to ensure sufficient regional concentrations while preventing excitotoxic damage from excess glutamate at the synaptic cleft (Sullivan et al., 2007). However, during periods of excitotoxic stress, such as occur with neuroinflammation, glutamate concentrations are increased and any drug-induced reduction in GFAP, and thus GFAP-anchored glutamate transporters, would have enormous impacts in exacerbating neurologic injury. Furthermore, GFAP is an important structural protein in astrocyte reactivity to neurologic insult and astrogliosis during recovery. Because intensive care exposures are prolonged and often require weaning, it is not just the acute drug effects that require attention, but also those during convalescence that might impair recovery.

While benzodiazepines are most often singled out due to their close association with clinical delirium, opioid medications are ubiquitous in intensive care and as noted previously, also have important neuropathologic effects. Relevant to critical illness, which is typified by endothelial activation and inflammation, opioids bind to microglial Toll-Like Receptor 4 (TLR-4) and have been shown to induce neuroinflammation in a manner similar to gram negative bacterial lipopolysaccharide (Wang et al., 2012). This may explain the observation by van Zellem et al.’s (2014) that children who survived meningococcemia were more likely to score lower on IQ tests several years later if they received any kind of opioid compared to children who received other sedatives. Interestingly, astrocytes also express TLR-4, and are important participants in, and modulators of, neuroinflammation through their expression of S100 calcium-binding protein (S100B) (Bowman et al., 2003; Van Eldik and Wainwright, 2003; Li et al., 2020). While opioids are typically associated with neuroinflammation through TLR4, Iqbal O’Meara et al. (2020) found a significant increase in cerebral S100B levels in childhood-approximate rats treated with benzodiazepine. Exacerbated neuroinflammation may be a key mechanism by which sedatives contribute to intensive care neurologic morbidity, and because cholinergic signaling is anti-inflammatory, the anticholinergic activity of some opioids and sedatives needs to be considered as well.

In addition to modulating neuroinflammation, exogenous opioid medications might affect endogenous opioid pathways that are involved in myelination. This certainly impacts neurodevelopment, but also affects myelin integrity and remyelination during recovery. Sanchez et al. (2008); Eschenroeder et al. (2012), and Vestal-Laborde et al. (2014) have found dose-dependent effects of opioid medications on developmental myelination, with lower doses accelerating oligodendrocyte maturation and myelin compaction in prenatal rodents, and higher doses having an opposite inhibitory effect that involves the nociceptin/orphanin FQ receptor. Because nociceptin is involved in pain signaling, this effect may have broader implications for the development of hyperalgesia during critical illness, as the Sato-Bigbee group has also reported changes in glutamate transporters with increased nociceptin receptor activation (Meyer et al., 2017). Interestingly, Iqbal O’Meara et al. (2020) did not find that opioid treatment of early childhood-approximate rats changed the expression of myelin basic protein (MBP), but treatment with benzodiazepine did significantly increase MBP levels. Despite evidence that PICU-typical sedation could impact inflammation and myelin integrity in the context of links between white matter tract abnormalities and delirium and post-discharge cognitive dysfunction, there remains insufficient investigation into the myelin effect of PICU-typical sedation at a variety of ages in a variety of disease entities. This is of particular interest because opioids are independently associated with delirium and post-intensive care syndrome, and they have the compounded potential to affect neuroinflammation, synaptic plasticity and neurotransmission, and myelin integrity.

### Representative Models of PICU-Typical Neurologic Stress and Sedation Are Needed

It is easy to conclude from a large body of work that drug-induced neuronal death, synaptic derangement, dysmyelination, or altered neurotransmitter trafficking results in cognitive dysfunction after critical illness, but this assumes that these drugs have independent effects that are unrelated to developmental stage and/or the surrounding physiology. To date, the bulk of this work has been performed in healthy models with insufficient investigation of multidrug sedation combined with PICU-typical brain stress: ischemia, acidosis, hypoxemia, and inflammation. Lines of investigation are also lacking into the effects of sedatives and analgesics on the evolution of common disease states such as TBI, stroke, cardiac arrest, and sepsis, as well as effects on recovery from them. Brain development is dynamic and delirium and neurologic dysfunction are bimodal, with increased prevalence concentrated at either end of the spectrum: immaturity of early childhood and senescence of the geriatric adult. Importantly, while age and illness factors cannot be changed, analgesia and sedation are potentially modifiable, and a better understanding of the discrete contribution of these drugs in the larger context of critical illness is sorely needed.

**FIGURE 4:**
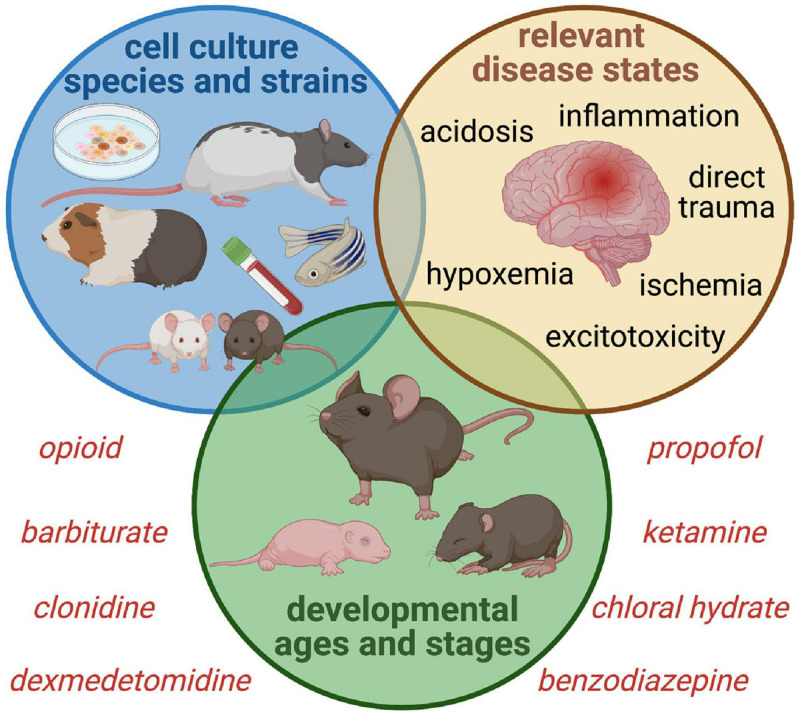
Proposed Experimental Paradigms for Intensive Care Sedation and Neurologic Sequelae. Studies of sedative and analgesic use during critical illness must account for the complex interaction between drug altered neurotransmission and a wide variety of disease states that alter normal neurophysiology, ranging from primary brain injury to neurologic stressors such as hypoperfusion and inflammation. Sex and age-dependent vulnerability must be considered, as well as a variety of phenotypes to ensure generalizability of findings. Incorporating clinically relevant biomarkers bolsters translational value as well.

As shown in Figure 4, a “reverse translational” approach is needed in order to replicate critical illness so that more accurate correlations can be drawn and safer sedation strategies can be developed. Intensive care sedation is seldom to never achieved with a single drug, and while single drug research is vital to understanding the role of each pathway, the effect of drug combinations is much more relevant. Until we have more representative models to elucidate what happens in the brain of a septic infant treated for 2 weeks with opioid and benzodiazepine, for example, we are left extrapolating data from healthy animal models which may be inaccurate or misleading. In addition, models that clarify the mitigating or exacerbating effect of sedatives during neurologic injury or neurocritical illness are vital; it is improbable that any of these drugs have identical effects during health and illness.

Delirium and PICS-p symptoms do not discriminate, and uniformity of laboratory subjects is not translatable to the heterogeneous PICU population. The use of exclusively male or female animals should be discouraged, as there are important sex effects to be considered. A variety of developmental stages is also important, as effects may be age-dependent. For example, the perinatal and infant brain relies on inherently active astrocytes and microglia that may be more easily triggered into hyperinflammatory responses, and several growth factors and cytokines that are involved in early brain development are also pro-inflammatory (Deverman and Patterson, 2009). Likewise, establishing whether experimental effects are generalizable across more than one strain of mouse or rat or outbred stock may be informative as there are traits inherent to different strains making them more or less favorable for one type of research or another. Finally, the starkness of the laboratory vivarium should be strongly considered when translating cognitive effects from learning and memory in animals that are far removed from the environments they evolved to function in. This has salient implications beyond the humane care of these animals or the risk of anthropomorphism. Human children experience robust environmental stimuli, and non-standard enriched vivarium housing has been shown by Konkle et al. (2010); Connors et al. (2015), and Kentner et al. (2016) to mitigate inflammation and improve stress response and behavioral testing performance in animal models. This type of effect impedes the translation of behavioral and learning and memory data from standardly housed animals to humans and suggests a reconsideration of animal housing to facilitate rigorous results. Alternatively, this type of data could be used to justify early childhood intervention or rehabilitation in survivors of childhood critical illness. Investigators should consider the characteristics and natural behaviors and tendencies of their model species when evaluating cognition in order to have clarity on what they are actually measuring (de Waal, 2017; Genzel, 2021). Poor performance on learning and memory tasks may be attributable to motivation, mood, or sensorimotor deficits rather than cognition in the colloquial sense (Arakawa, 2019). On the other hand, this may be translatable to the current challenge of directly measuring cognitive deficits in children through full neuropsychological testing versus assessing school performance which is impacted by functional and social cognition.

## Discussion

While clinical evidence of anesthetic neurotoxicity is less definitive than animal models would suggest, clinical studies of neurologic dysfunction in critically ill children are far more convincing but with a paucity of translational basic science. In addition, while post-PICU outcome research has blossomed in the last decade, cognitive outcome studies are hindered by a lack of objective diagnostic criteria that are easy to implement. Moreover, neurodevelopment is dynamic, and evaluations need to be age-appropriate with sufficient follow-up intervals. Isolating the mechanistic contribution of sedatives is challenging in the complex milieu of intensive care, yet available multivariable studies continue to single out sedatives and opioid analgesics as risk factors. The exact role of these medications is poorly understood whether they are directly neurotoxic, as laboratory evidence would suggest in healthy animals, or whether this role shifts in the midst of hypoxemia, hypoperfusion, inflammation, acidosis, excitotoxicity, and/or sleep deprivation. It may be that certain drugs are more detrimental in specific disease states, and studies helping to differentiate between patient vulnerabilities in certain critical illnesses could help providers make more informed choices regarding sedation and analgesia. To that end, large scale collaboration and standardization of post-PICU cognitive assessment is needed in order to tease out the interactive components of this complex matrix.

There is momentum in the long-term follow-up of survivors of childhood critical illness, and multicenter collaborations are doing so with data-sharing in order to move the field forward: Pediatric Outcomes Studies after PICU Investigators (POST-PICU), Pediatric Acute Lung Injury and Sepsis Investigators (PALISI), Pediatric Neurocritical Care Research Group (PNCRG), the Collaborative Pediatric Critical Care Research Network (CPCCRN), and Pediatric Intensive Care Optimizing Long-term Outcomes (PICOLO). Members of POST-PICU and CPCCRN recently undertook a scoping review of the post-PICU discharge outcomes literature in order to facilitate consensus as the specialty tackles this important problem. In order to enroll and follow-up as many survivors as possible to accurately gauge the scope of cognitive dysfunction, a balance needs to be struck between ease of assessment (such as proxy metrics that can be obtained over the phone) and the thoroughness of a full neuropsychiatric battery. Prospective studies that account for sedation exposure are needed, along with age-appropriate metrics that accurately assess not only cognition, but also factors important to functional success, such as sleep and school performance.

Current practice shifts to improve outcomes are toward reducing total sedative exposure, daily sedation interruptions, increasing day/night orientation and promoting sleep, and incorporating non-pharmacologic means such as family presence and music to reduce stress and anxiety (Chlan et al., 2013). Early mobility initiatives are also on the rise, and have the benefit of increasing daytime orientation and activity for sleep at night, reducing immobility contractures and neuropathic pain, and possibly stimulating the production of protective neurotrophic factors. However, sedatives often cannot be avoided and one drug may be better than another in a given pathophysiology. For example, neuroinflammation has been shown to decrease glutamate transporter density and increase the ambient concentration of potentially excitotoxic glutamate (Tilleux and Hermans, 2007; Takaki et al., 2012). Partial blockade of NMDAR may therefore be beneficial, but we do not know which patients would benefit or when in the course of illness this would be effective. We also do not know if this should be avoided in a particular age group, disease state, or altogether, as NMDAR antagonists have been shown to increase neuronal death during early brain development in animal models. The risk/benefit ratio of NMDAR antagonism may be entirely different during critical illness brain stress or injury, but vital evidence for this type of intentional use of sedative drugs is lacking from representative animal models.

In conclusion, the impact of sedatives and analgesics in pediatric critical illness is a burgeoning field, but one with far-reaching implications in longitudinal recovery. Cognitive morbidity is a particularly understudied area of PICS-p in children, and the domain most likely impacted by sedatives and analgesics as shown in animal models and pediatric anesthesia literature. We need a better understanding of the impact of prolonged and repeated exposure to neuroactive medications on the developing brain, particularly during PICU admission with concurrent brain injury, sleep disruption, and neurologic stress. This will require appropriately designed studies testing physiologically derived hypotheses with age and domain appropriate outcome measures. This understanding is paramount to designing meaningful interventions. We need a translational research bridge to determine the right medication for the right disease in the right child at the right time to ensure the best possible recovery for our patients.

## Author Contributions

AT co-wrote the abstract, sections “Introduction” and “Sleeping With One Eye Open,” and created Figures 2, 3. TS co-wrote the section “Is a Better Mouse(Rat)Trap the Answer?”. KD co-wrote the section “Cognitive Dysfunction.” TH co-wrote and revised the section “Cognitive Dysfunction” and co-created Figure 1. CW co-wrote the sections “Introduction,” “Cognitive Dysfunction,” “Sleeping With One Eye Open,” and “Discussion,” revised the manuscript, and co-created Figure 1. KG co-wrote the sections “Introduction” and “Cognitive Dysfunction,” revised the manuscript, and co-created Figure 1. SM co-wrote the section “Cognitive Dysfunction” and revised the manuscript. AI originated the manuscript and invited co-authors, co-wrote all the sections, revised the manuscript in its entirety, and created Figure 4. All authors have given approval for publication and agreed to be accountable for its accuracy and integrity.

## Author Disclaimer

The opinions and assertions expressed herein are those of the authors and do not necessarily reflect the official policy or position of the Uniformed Services University or the Department of Defense. The content is solely the responsibility of the authors and does not necessarily represent the official views of the National Institutes of Health.

## Conflict of Interest

The authors declare that the research was conducted in the absence of any commercial or financial relationships that could be construed as a potential conflict of interest.

## Publisher’s Note

All claims expressed in this article are solely those of the authors and do not necessarily represent those of their affiliated organizations, or those of the publisher, the editors and the reviewers. Any product that may be evaluated in this article, or claim that may be made by its manufacturer, is not guaranteed or endorsed by the publisher.
